# Luminescent Complexes of Europium (III) with 2-(Phenylethynyl)-1,10-phenanthroline: The Role of the Counterions

**DOI:** 10.3390/molecules26237272

**Published:** 2021-11-30

**Authors:** Denitsa Elenkova, Rumen Lyapchev, Julia Romanova, Bernd Morgenstern, Yana Dimitrova, Deyan Dimov, Martin Tsvetkov, Joana Zaharieva

**Affiliations:** 1 Faculty of Chemistry and Pharmacy, Sofia University, 1164 Sofia, Bulgaria; ohrl@chem.uni-sofia.bg (R.L.); fhjr@chem.uni-sofia.bg (J.R.); yanadim123@gmail.com (Y.D.); nhmt@chem.uni-sofia.bg (M.T.); nhjz@chem.uni-sofia.bg (J.Z.); 2 Department of Inorganic Solid-State Chemistry, Saarland University, 66123 Saarbrücken, Germany; bernd.morgenstern@uni-saarland.de; 3 Institute of Optical Materials and Technologies, Bulgarian Academy of Science, 1113 Sofia, Bulgaria; dean7110@gmail.com

**Keywords:** lanthanoid, bidentate ligands, X-ray diffraction, fluorescent spectroscopy, DFT/TD-DFT and TDA

## Abstract

New antenna ligand, 2-(phenylethynyl)-1,10-phenanthroline (PEP), and its luminescent Eu (III) complexes, Eu(PEP)_2_Cl_3_ and Eu(PEP)_2_(NO_3_)_3_, are synthesized and characterized. The synthetic procedure applied is based on reacting of europium salts with ligand in hot acetonitrile solutions in molar ratio 1 to 2. The structure of the complexes is refined by X-ray diffraction based on the single crystals obtained. The compounds [Eu(PEP)_2_Cl_3_]·2CH_3_CN and [Eu(PEP)_2_(NO_3_)_3_]∙2CH_3_CN crystalize in monoclinic space group P2_1/n_ and P2_1/c_, respectively, with two acetonitrile solvent molecules. Intra- and inter-ligand π-π stacking interactions are present in solid stat and are realized between the phenanthroline moieties, as well as between the substituents and the phenanthroline units. The optical properties of the complexes are investigated in solid state, acetonitrile and dichloromethane solution. Both compounds exhibit bright red luminescence caused by the organic ligand acting as antenna for sensitization of Eu (III) emission. The newly designed complexes differ in counter ions in the inner coordination sphere, which allows exploring their influence on the stability, molecular and supramolecular structure, fluorescent properties and symmetry of the Eu (III) ion. In addition, molecular simulations are performed in order to explain the observed experimental behavior of the complexes. The discovered structure-properties relationships give insight on the role of the counter ions in the molecular design of new Eu (III) based luminescent materials.

## 1. Introduction

Trivalent lanthanoid (Ln (III)) complexes with organic ligands are promising materials for a wide range of applications such as sensors for small molecules (cations, anions, pH sensors) or chemical pollutants [1,2,3], luminescent bioprobes [4,5], organic-light emitting diodes (OLEDs) [6,7], in time-resolved microscopy [8,9], coating for photovoltaics and anticancer agents [10,11,12,13,14]. The molar absorption coefficient of the trivalent lanthanoid ions are very small (about 0.1–10 M^−1^cm^−1^) because the f-f transitions are Laporte-forbidden and if the samples are directly excited this result in low luminescence intensity [15]. A strategy to overcome this problem is proposed by Weissman et al. [16], who discovered that organic ligands can absorb light and transfer it to the emitting levels of the Ln (III) ions. The energy transfer from the organic chromophore to the metal is intramolecular and significantly enhances the luminescence intensity. Such photophysical process of Ln (III) ions sensitization is often referred as antenna effect. Due to the wide application potential of lanthanoid complexes and the unique antenna effect their photoluminescence properties have been extensively studied in solution [17,18,19], sol gel glasses [20,21], ionogels [22], liquid crystals [23], polymer matrices and into inorganic hosts or in metal-organic frameworks (MOFs) [24,25,26].

Aromatic N-donor ligands such as 1,10-phenanthroline can be altered by substituting different groups in the heterocyclic moieties, among those phenyl, methylphenyl, dimethylphenyl, phenylethynyl [27], ethynylpyridine [28], just to mention a few. The phenanthroline and its derivatives are known for being ligands for different metal ions as well as effective photosensitizers of lanthanoids [27]. Therefore, a number of europium (III) complexes with phenanthroline-based antenna have been reported recently [29,30,31]. Since the antenna effect is extremely interesting from fundamental point of view, many theoretical and spectroscopic studies discussed the photophysical process of absorption of light, the excited state energy transfer from the ligand to the europium ion and the luminescent properties of these compounds [30,31,32,33,34]. Application-oriented investigations on phenanthroline-based complexes also demonstrated their importance for the advance technologies [35,36,37,38,39,40].

**Figure 1:**
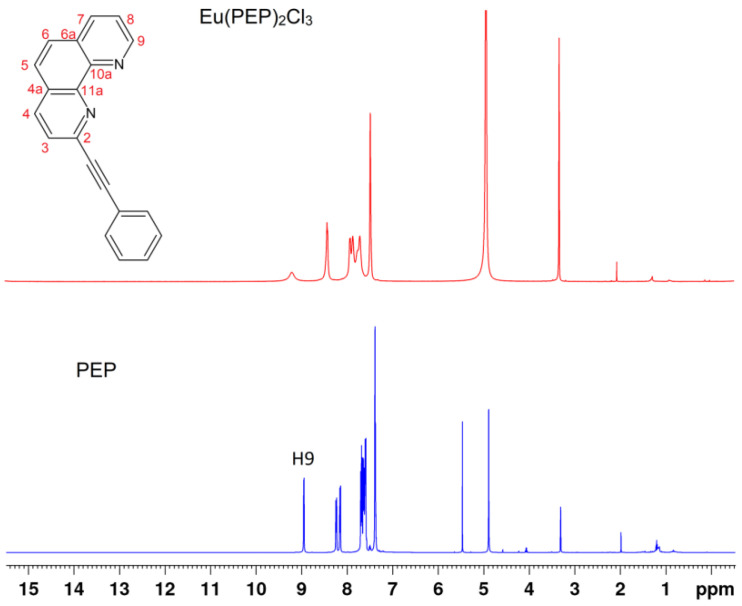
^1^H-NMR spectra of the 2-(phenylethynyl)-1,10-phenanthroline (PEP) ligand and Eu(PEP)_2_Cl_3_ complex in MeOD.

**Figure 2:**
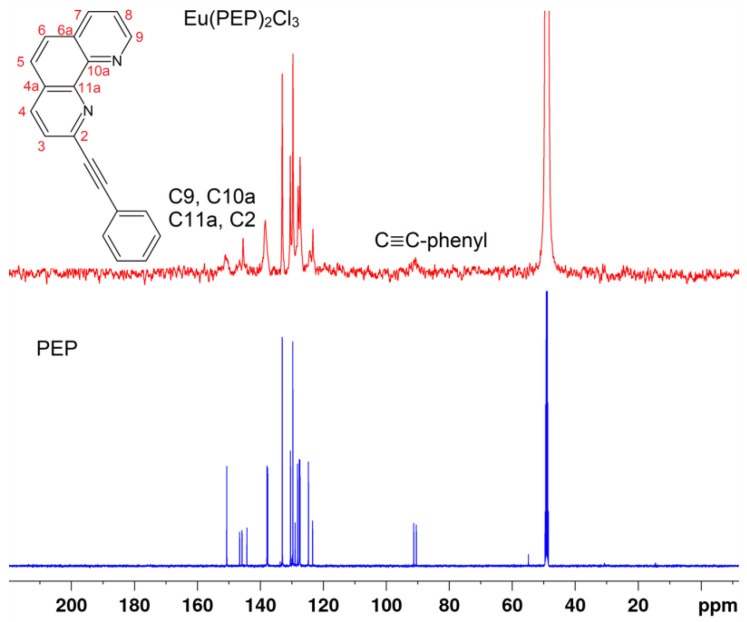
^13^C-NMR spectra of PEP ligand and Eu(PEP)_2_Cl_3_ complex in MeOD.

Despite the enviable scientific interests, the large number of papers and the accumulated knowledge on complexes of Eu (III) with phenanthroline type of ligands, there is still a room for improvement in the fundamental understanding of the photophysical properties of these compounds, as well as in the strategies for their design for application purpose. For example, a number of articles are paying attention to the crystal structure of Eu (III) complexes with phenanthroline and different counter ions as nitrates, chlorides, thiocyanates, carboxylates anions [41,42,43,44,45,46]. However, as most of the complexes of lanthanoids with phenanthroline are with poor solubility in solvents usually used in spectroscopy, their photophysical properties are mostly studied in solid state [29,30] and there is limited information on the influence of different counter ions on the energy of the ligand-based triplet excited state, the photophysical properties and the stability of the complexes. The present work aims to give insights on these questions by design of two new structurally similar complexes of europium (III) with 2-(phenylethynyl)-1,10-phenanthroline (PEP) and nitrates or chlorides as inorganic ligands: [Eu(PEP)_2_Cl_3_]·2CH_3_CN and [Eu(PEP)_2_(NO_3_)_3_]·2CH_3_CN (Figure 1 and Figure 2).

The reasons for choosing the chemically modified phenanthroline as an antenna ligand for europium (III) sensitization are as follow: (i) the substitution in heterocycles will modulate the energy of the ligand-based excited triplet state and hence the luminescent properties of the complexes and (ii) the functionalization with 2-(phenylethynyl) group will improve the solubility of the compounds in organic solvents, which is very important factor from application point of view. By using this strategy, we were able to obtain and characterize the photophysical behavior of the new phenanthroline-based Eu (III) complexes in solid state, as well as in acetonitrile and dichloromethane (DCM) media. Moreover, due to the presence of the 2-(phenylethynyl) substituents the novel Eu (III) complexes show intra- and intermolecular π-π stacking between the PEP ligands, which is believed to facilitate the supramolecular ordering and to serve as an approach for crystal engineering. Theoretical simulations on the structure and optical properties of the newly synthesized complexes were also performed aiming deeper understanding of the structural and environmental factors that govern the luminescent response. Comparison between experimental and theoretical results allowed us to reveal the role of the counter ions on the photophysical properties of the complexes, as well as on their stability in solvent media-effects, which can be directly translated into material design guidelines.

## 2. Results and Discussion 

### 2.1. Characterization of Polycrystalline Samples

The lanthanide content of the complexes was determined thermogravimetrically by calcination of the complex up to 1300 °C for 4 h, taking into account that the powder residue formed was Eu_2_O_3_. The mass of residue was measured by a digital analytical weighing scale with 1 × 10^−5^ g accuracy. The content of Eu, %, was 18.55 experimental/18.56 calculated for the complex Eu(PEP)_2_Cl_3_ as well as 17.10 experimental/16.91 calculated for Eu(PEP)_2_(NO_3_)_3_. The metal content is corresponding well with the composition of the complexes where the ratio metal:ligand:counterion is equal to 1:2:3. No evidence of solvent was observed which is correlating well with TG/DTA analysis and NMR results. In TG/DTA curves of both complexes (Figure S1) there are no evidences of endothermic picks in the range of 70–100 °C, where acetonitrile could be expected. Also in ^1^H–NMR spectrum of Eu(PEP)_2_Cl_3_ (Figure S2) there are only traces of acetonitrile and from the integration values it is clear that it is not significant amount. Integration of proton shift in the spectrum of the complex corresponds exactly to the number of protons we expect to see. The presence of water shift is due to some traces of water in MeOD and it is also visible in the spectrum of PEP ligand (Figure 1). In ^13^C-NMR spectrum of the complex (Figure S3) no any acetonitrile carbons or some other impurities are noticeable.

Comparisons of NMR spectra of PEP ligand and Eu(PEP)_2_Cl_3_ in Figure 1 and Figure 2, reveal clearly the coordination of Eu (III) to the ligand as all protons and carbons related to the coordination are highly influenced. For example, proton H9 is the closest proton to the coordination in Figure 1 and carbons C9-phenanthroline, ^4^C11a-phenanthroline, ^4^C10a-phenanthroline, ^4^C2-phenanthroline (Figure 2). It is interesting to point out that C≡C-phenyl, C≡C-phenyl carbons are also influenced strongly by coordination. For assignment of the shift in the ligand, please see subsection it should be For assignment of the shift in the ligand, please see Section 4.2.1. Synthesis of the 2-(Phenylethynyl)-1,10-phenanthroline Ligand.

**Figure 3:**
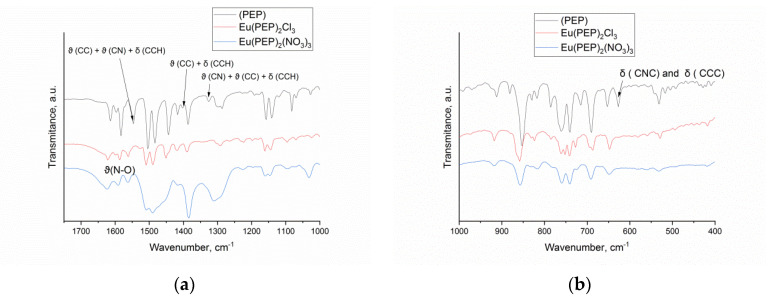
The experimental IR spectra of the PEP ligand, and the complexes in KBr pellets (from top to bottom) in the interval (**a**) 1750–1000 cm^−1^ and (**b**) 1000–400 cm^−1^.

The assignment of the IR bands, as well as a detailed comparison between the experimentally observed and simulated vibrational frequencies ω [cm^−1^] for the PEP ligand and the complexes can be found in the SM, Table S1. There is an overall good agreement between theory and experiment, except for the stretching of the (-C≡C-) triple bond. Both approaches suggest similar qualitative changes in the IR spectrum of the PEP after complexation. Coordination of the PEP to the Eu (III) causes a shift in most of the IR bands of the ligand to higher frequencies and the change in four key IR bands can be suggested as a signature of the complexes formation: The first signature of complexation is the IR band associated with C-C and C-N stretching, as well as CCH bending in the PEP ligand (1546 cm^−1^), which shifts with 16/17 cm^−1^ in the complexes. The second signature of complexation comes from a normal mode representing C-C stretching and CCH bending in the PEP ligand (1402 cm^−1^), which shifts with 15 cm^−1^ after the formation of the metal-ligand bonds (Figure 3a). The third and fourth signatures of complexation are in the lack of IR peaks around 1326 and 627 cm^−1^ in the complexes, while in the PEP ligand such bands are detectable and correspond to the C-N and C-C stretching, combined with CCH bending (1326 cm^−1^ ), as well as to CNC and CCC bending (627 cm^−1^) seen in Figure 3b. It also worth to note that the IR spectra of both complexes are very similar and the only difference is the presence of the counterions signatures for the Eu(PEP)_2_(NO_3_)_3_, while the Cl^-^ ions are found to be IR active below 500 cm^−1^, i.e., below the experimentally scanned region. 

Solubility and behavior of the complexes in different solvent were investigated. It was established that the solutions of the complexes in some organic solvents such as alcohols (ethanol, methanol, isopropanol), DMSO, and DMF did not exhibit red luminescence. Considering the solubility of the complexes Eu(PEP)_2_Cl_3_ and Eu(PEP)_2_(NO_3_)_3_ in acetonitrile it was limited to 1.5 mg/10 mL for Eu(PEP)_2_Cl_3_ and 3 mg/10 mL for Eu(PEP)_2_(NO_3_)_3_. The solubility in dichloromethane and chloroform was even lower but practically enough to perform measurements in solution. It was not detected any solubility of the complexes in diethyl ether and some hydrocarbons tested (hexane, heptane, cyclohexane).

### 2.2. Crystal Structure 

**Figure 4:**
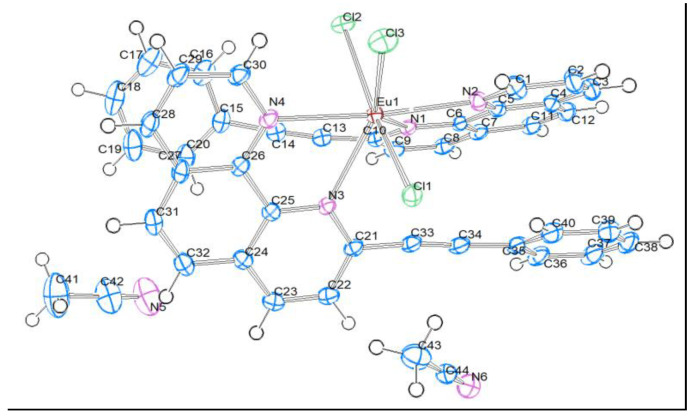
Structure of Eu(PEP), thermal ellipsoid plots (50% probability contours) of the structure.

The [Eu(PEP)_2_Cl_3_]·2CH_3_CN complex (referred in the text below as Eu(PEP)_2_Cl_3_) crystallizes in the monoclinic space group P2_1/n_ (Figure 4). The asymmetric unit consists of one neutral [Eu(PEP)_2_Cl_3_] complex in a general position and two CH_3_CN solvent molecules. The Eu (III) is seven-coordinated by four N atoms from two 2-(phenylethynyl)-1,10-phenanthroline ligands and three Cl^−^ ions completing the neutral charge of the complex forming a distorted heptagon. The bond lengths between Eu and N are in the range from 2.5320(13) to 2.5822(14) Å and the Eu–Cl bond lengths are in the range of 2.6744(5)–2.6830(5) Å (Table 1).

**Figure 5:**
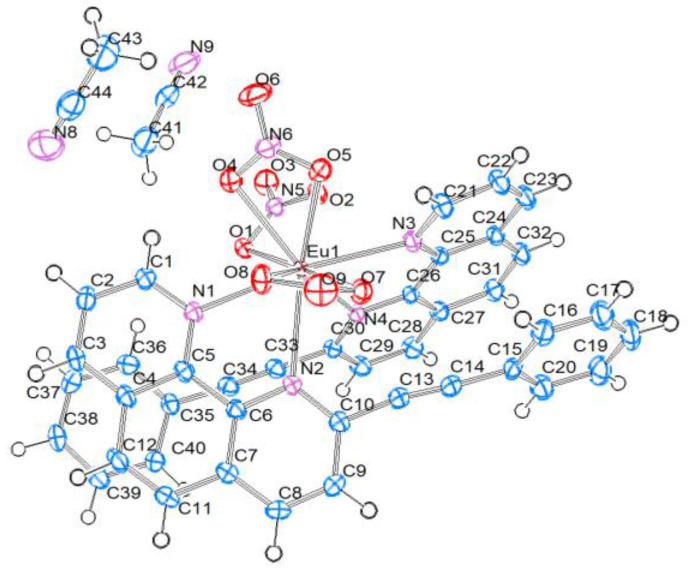
Structure of Eu(PEP)_2_(NO_3_)_3_, thermal ellipsoid plots (50% probability contours) of the structure.

The [Eu(PEP)_2_(NO_3_)_3_]·2CH_3_CN compound (referred in the text below only as Eu(PEP)_2_(NO_3_)_3_) crystallizes in the monoclinic space group P2_1/c_ with asymmetric unit consisting of one neutral [Eu(PEP)_2_(NO_3_)_3_] molecule in a general position and two CH_3_CN solvent molecules (Figure 5). The central metal ion is ten-coordinated by four N atoms from two 2-(phenylethynyl)-1,10-phenanthroline ligands and three bidentate nitrate ions. The Eu–N bond lengths are slightly higher than in the Eu(PEP)_2_Cl_3_ complex and range from 2.5958(15) to 2.6542(15) Å due to steric interaction between the organic ligands and the three nitrate ions. The bond lengths between Eu and O atoms of the nitrate ions are in the range of 2.4813(13)–2.5273(14) Å (Table 1).

**Figure 6:**
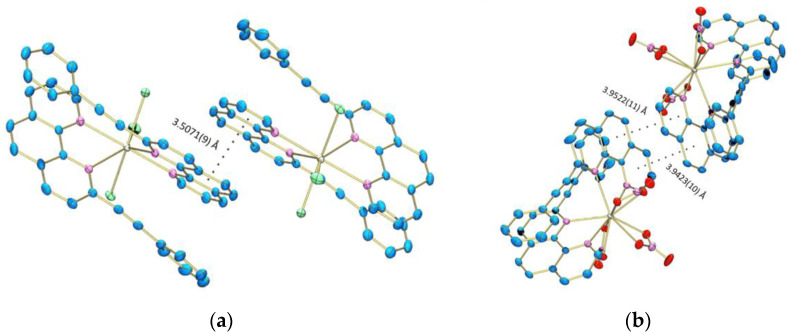
Intermolecular π–π interactions in (**a**) Eu(PEP)_2_Cl_3_ and (**b**) Eu(PEP)_2_(NO_3_)_3_.

**Figure 7:**
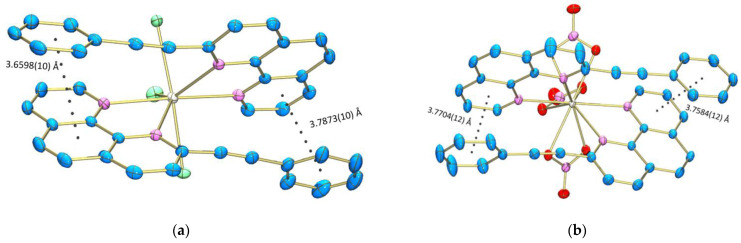
Intramolecular π–π interactions of (**a**) Eu(PEP)_2_Cl_3_ and (**b**) Eu(PEP)_2_(NO_3_)_3_.

In both crystals no classical hydrogen bonding interactions were found, but the crystal packing is stabilized by inter- and intramolecular π–π stacking interactions between the neighboring PEP ligands (Figure 6 and Figure 7). The ligand-ligand distances, slippage d[a] and dihedral angle between the planes are presented in Table S2. The computational results reveal similar thermodynamic stability of the Eu(PEP)_2_(NO_3_)_3_ complex in acetonitrile and in DCM solution. The Eu(PEP)_2_Cl_3_ compound on the other hand is ~1 kcal/mol more stable in acetonitrile than in DCM solution. Therefore, it can be speculated that the experimentally observed instability of the complex Eu(PEP)_2_Cl_3_ in DCM has a thermodynamic origin. Specific intermolecular interactions between the solvent and the solute may additionally increase the instability of Eu(PEP)_2_Cl_3_·DCM.

The complex Eu(PEP)_2_Cl_3_ is characterized with strong intermolecular π–π inter-actions between the adjacent PEP ligands with almost no slippage (0.616/0.715 Å) and 3.51 Å between the centroids of interacting rings. In this case, the aromatic-aromatic interaction is realized between the phenanthroline part of the ligands. The presence of π–π interactions leads to significant stabilization of the crystal structure of Eu(PEP)_2_Cl_3_ compared to the structure of Eu(PEP)_2_(NO_3_)_3_ complex, for which longer intermolecular π–π stacking distances are observed. This finding is well supported by the TG/DTA analysis shown on Figure S1, which reveals greater thermal stability of the Eu(PEP)_2_Cl_3_ complex. The Eu(PEP)_2_Cl_3_ complex is stable up to 365 °C, while the Eu(PEP)_2_(NO_3_)_3_ complex up to 230 °C. Furthermore, the TG curve of the Eu(PEP)_2_(NO_3_)_3_ complex shows stepwise thermal decomposition probably related to separate thermal oxidation of both ligands, which indicates weak interaction between them. The first step occurs from 230 until 410 °C, followed by complete thermal oxidation.

Intramolecular interactions are also present in both complexes and more specifically, they are realized between the phenyl ring of the substituent in one of the ligand and the phenanthroline area of the other ligand. The intramolecular π–π stacking distances are about 3.66 Å and 3.76 Å for Eu(PEP)_2_Cl_3_ and Eu(PEP)_2_(NO_3_)_3_, respectively. Therefore, interestingly enough it can be concluded that the interactions in Eu(PEP)_2_Cl_3_ are stronger compared to the Eu(PEP)_2_(NO_3_)_3_. Major role for these differences is credited to the size of the inorganic ligands with NO_3_**^−^** being much larger compared to Cl^−^. All this leads to significant distortion of the geometry of the complexes and their thermal stability.

The X-ray single-crystal measurements explain the structure of the complexes in solid state and in order to investigate the geometry of the complexes in solution we have performed theoretical simulations. The results for the ground state structure of the complexes obtained with the DFT approach in implicit solvent environment are presented in Table 2. It is revealed that in the solution both complexes are symmetric with respect to both antenna ligands. In addition, the nitrogen atoms from the functionalized pyridine ring of the phenanthroline moiety (N^≡^) form weaker bonds with the metal than the nitrogen atoms of the non-functionalized pyridine ring (N) of the ligand. In implicit solvent environment, quantitative differences in the metal-ligand bond lengths as a function of the anions are observed. Namely, in the Eu(PEP)_2_(NO_3_)_3_ complex the Eu-N bonds are shorter and the Eu-N^≡^ distances are longer than in the case of the Eu(PEP)_2_Cl_3_ complex (the bond length difference between the compounds is about +/− 0.01–0.02 Å). Here, it is important to note that the DFT simulations suggest that in solvent the intramolecular π-π interaction may be retained. The intraligand distance between the phenyl substituent and the central ring of phenanthroline in implicit solvent environment vary as follows: Eu(PEP)_2_Cl_3_ 3.29–3.90 Å and Eu(PEP)_2_(NO_3_)_3_ 3.48–3.72 Å. The π-π overlap is better for the Eu(PEP)_2_(NO_3_)_3_ complex, while slight rotation is observed for the Eu(PEP)_2_Cl_3_ compound.

To some extend quantitative and qualitative differences between the theoretically predicted structure in implicit solvent and the experimentally observed geometry in solid state exists and this can be explained by the different environment. Such differences are expected especially when taking into account the role of the π-π stacking interactions for the crystal structure formation. Nevertheless, both theoretical and experimental approaches demonstrate that the counter ions may lead to structural changes in very similar europium (III) complexes.

### 2.3. Optical Properties 

**Figure 8:**
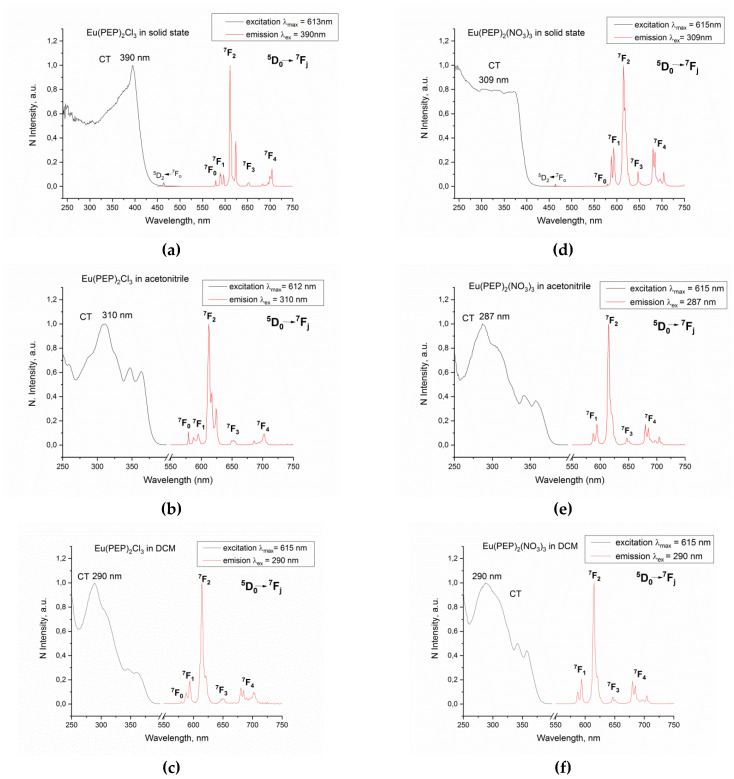
Luminescence spectra (corrected and normalized) of Eu(PEP)_2_Cl_3_ in (**a**) solid state, (**b**) acetonitrile solution, (**c**) DCM solution and of Eu(PEP)_2_(NO_3_)_3_ in (**d**) solid state, (**e**) acetonitrile solution, (**f**) DCM solution. All measurements are performed at 298 K.

The excitation and emission spectra of the complexes Eu(PEP)_2_Cl_3_ and Eu(PEP)_2_(NO_3_)_3_ in solid state, acetonitrile and DCM solution are presented in Figure 8. Upon excitation with UV light both compounds emit characteristic red light due to the metal-centered ^5^D_0_ => ^7^F_J_ (J = 0–4) transitions. The excitation spectra correspond to the ligand absorption spectra (Figure 8, Figures S4 and S5) and hence confirm the antenna effect. Emission from the free ligand is also observed in solid state and in solution with maximum about 420 nm and 400 nm, respectively (Figures S6 and S7).

The excitation spectra of the complexes in solid state (Figure 8a,d) are slightly different. Sharp maximum in the spectrum of Eu(PEP)_2_Cl_3_ around 390 nm can be seen. The same band is also present in the excitation spectrum of Eu(PEP)_2_(NO_3_)_3_, but it is not that significantly distinctive and its excitation can be performed in range of about 300 to 400 nm without leading to a noteworthy change in the emission. The Eu(PEP)_2_(NO_3_)_3_ complex exhibits very long lifetime of the excited state of about 1.3 ms in solid state (Table 3), which is higher than the values for europium complexes with phenanthroline derivates reported by Maouche et al. and Akerboom et al. [29,30].

Even though the limited solubility of the compounds in acetonitrile media, the luminescence response of the complexes stored in a sealed container was stable. It was observed that the complexes lost their luminescence when kept in an open vessel exposed to air for several days. Therefore, we could assume that the acetonitrile-complex solution presents oxygen sensitivity properties. This observation could be interesting for further investigations due to the possibility of applying the complexes as oxygen sensors.

As in solid state, the Eu(PEP)_2_(NO_3_)_3_ complex shows more intensive luminescence. Considering the lifetime of the excited state, it is much higher for the nitrate than for the chloride containing complex. The emission quantum yields (QY) for both complexes in acetonitrile differ only with about 1% (Table 3). The emission lifetime for Eu(PEP)_2_(NO_3_)_3_ is higher than available experimental data for similar complexes in acetonitrile [47].

In freshly prepared DCM solution the luminescence behavior of complex Eu(PEP)_2_Cl_3_ significantly differs from that in solid state and acetonitrile solution. For example, in DCM the emission from Eu(PEP)_2_Cl_3_ decays with time even in a sealed container. The luminescence spectrum is changed compared to its luminescence spectra in solid state and acetonitrile solution (Figure 8a–c). Luminescence coming from the ligand as well as from the Eu (III) is registered (Figure S8). A change of the splitting of the ^5^D_0_ => ^7^F_J_ (J = 0–4) transitions is observed (Figure 8c) suggesting a change in the symmetry of the Eu (III) ion and an instability of Eu(PEP)_2_Cl_3_ in DCM. Moreover, the absorption maximum in the excitation spectrum of the complex shifts to the blue region when going from acetonitrile to DCM suggesting once again changes in the metal environment. Such shift in the excitation spectrum of Eu(PEP)_2_Cl_3_ is inconsistent with simulations on the optical properties of the complexes and the ligand (Table S4) showing negligible solvatochromic effects.

In order to gain more insight on the instability of complex Eu(PEP)_2_Cl_3_ in DCM solution we performed free energy calculations for the formation of the complexes (Table S3). The computational results reveal similar thermodynamic stability of the Eu(PEP)_2_(NO_3_)_3_ complex both in acetonitrile and in DCM solution. The Eu(PEP)_2_Cl_3_ compound on the other hand is ~1 kcal/mol more stable in acetonitrile than in DCM solution. Therefore, it can be speculated that the experimentally observed instability of the complex Eu(PEP)_2_Cl_3_ in DCM has a thermodynamic origin. Specific intermolecular interactions between the solvent and the solute may additionally increase the instability of Eu(PEP)_2_Cl_3_ in DCM.

Contrarily to the case of Eu(PEP)_2_Cl_3_, the Eu(PEP)_2_(NO_3_)_3_ complex in DCM is emitting intensively and show stable in time luminescence when stored in a sealed container. Moreover, complex Eu(PEP)_2_(NO_3_)_3_ exhibits longer lifetime and higher QY in DCM than in acetonitrile (Table 3). The QY for Eu(PEP)_2_(NO_3_)_3_ in DCM is about 3 times higher than in acetonitrile and close to reported values on phenanthroline-based complexes [29,30,47].

Comparison between the relative luminescence intensity of the two complexes (Table S3) in solid state and both solutions clearly demonstrates the radical difference between acetonitrile and DCM solution for the Eu(PEP)_2_Cl_3_ complex. For this compound the 0→2 transition loses intensity (from 81.39 to 64.54%) and the 0→4 transition gains (from 7.08 to 19.73%) intensity in DCM compared to acetonitrile (Table S4). The relative luminescence intensities for Eu(PEP)_2_(NO_3_)_3_ represented in Table S3 do not show significant change going from acetonitrile solution to DCM.

**Figure 9:**
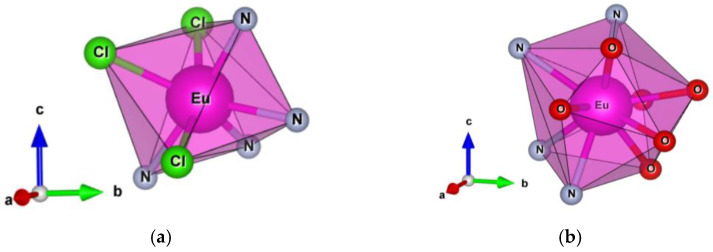
Capped trigonal prism geometry of Eu(PEP)_2_Cl_3_ (**a**); Sphenocorona geometry of Eu(PEP)_2_(NO_3_)_3_ (**b**).

The different stability and luminescent properties of Eu(PEP)_2_Cl_3_ in DCM solution can be explained with different coordination number and symmetry of the Eu (III) ion (Figure 9). The Eu (III) in complex Eu(PEP)_2_Cl_3_ has coordination number 7 with capped trigonal prism geometry (C2v) with the twofold axis going along the Eu1-Cl3 bond, while in complex Eu(PEP)_2_(NO_3_)_3_ the coordination number is 10 with sphenocorona geometry and C2 symmetry with the twofold axis along the Eu1-N6-O6 line of atoms as seen on (Figure 9) The splitting of the ^5^D_0_→^7^F_J_ transitions in the spectra of Eu(PEP)_2_Cl_3_ in solid state and acetonitrile (Figure 8a,b) is corresponding well to C_2v_ symmetry [48]. However, the splitting of the ^5^D_0_→^7^F_J_ transitions in DCM solution is more alike the one presented in all Eu(PEP)_2_(NO_3_)_3_ spectra and it corresponds to C_2_ symmetry. As well-known higher coordination numbers are preferable for Ln (III) ions and it is very important for the photophysical properties of the Ln (III) ions to be isolated from the influence of the solvent molecules leading to quenching effects on luminescence. In the case of Eu(PEP)_2_Cl_3_ complex, the DCM clearly interact with the Eu (III) ion, changing its symmetry and quenching the luminescence. Such possible interaction could be explained by is the additional coordination of DCM molecules to the Eu(PEP)_2_Cl_3_ complex. In DCM media there is no longer [Eu(PEP)_2_Cl_3_] and most probably DCM molecules are entering the inner coordination sphere which is an undesirable effect.

In order to understand the optical properties of the complexes and the PEP ligand in more details, we have performed quantum-chemical calculations in vacuum and implicit solvent environment. Results from the simulations are presented in Table 4. They demonstrate again that the absorption and emission properties of the complexes depend on the nature of the anionic ligands. At the TD-DFT/TDA level the vertical excitation and emission energies for Eu(PEP)_2_Cl_3_ are lower than in the case of Eu(PEP)_2_(NO_3_)_3_. Therefore, it can be concluded that although not directly involved in excitations, the anionic ligands can somehow affect the photodynamics in the system and the emission properties of the europium ion.

Comparison between the TD-DFT/TDA results for the complexes and for the isolated ligand clearly indicates that the key excited state T_1_ originates from the PEP ligand. According to the calculations, the ligand centered T_1_ states in Eu(PEP)_2_Cl_3_ and Eu(PEP)_2_(NO_3_)_3_ are located at ~2.45 eV and ~2.62 eV, respectively. Therefore, for both complexes the T_1_ states are about 0.3 eV higher than the emitting levels of the europium ion [48]: ^5^D_0_~2.14 eV and ^5^D_1_~2.35 eV, which confirms the antenna capacity of the PEP ligand. Based on these data it can be speculated that the mechanism of energy transfer in the complexes involves the ligand-based T_1_ state, as well as the ^5^D_0_ and ^5^D_1_ emitting states of the metal ion. The excited states energetics at the TD-DFT/TDA level suggests that higher rate of the backward energy transfer from the ^5^D_1_ level to the PEP ligand can be expected in Eu(PEP)_2_Cl_3_ due to the smaller energy difference between the states of interest than in the case of Eu(PEP)_2_(NO_3_)_3_.

## 3. Conclusions

The chemical modification of 1,10-phenanthroline by the substituent phenylethynyl on the 2-position designed new ligand 2-(phenylethynyl)-1,10-phenanthroline (PEP) which was used for synthesis of the new luminescent complexes of Eu (III), Eu(PEP)_2_Cl_3_ and Eu(PEP)_2_(NO_3_)_3_. A combined experimental and theoretical study on both complexes revealed they were structurally similar but differ in the type of the counterions, which allowed us to conclude on their role for the molecular and crystal structure, stability, Eu (III) symmetry, as well as for the luminescent properties of the compounds. Moreover, the presence of 2-(phenylethynyl) substituent in the ligand increased the solubility by that allowing analysis of their structure and properties, not only in solid state but also in DCM and acetonitrile solutions.

Both for polycrystalline samples and single crystals, the XRD was not corresponding to any structure in the crystal database and by that proved the novelty of Eu (III) complexes with 1,10-phenanthroline derivative. The X-ray measurements reveal that the use of smaller in size and monodentate Cl- counterions results in stronger interaction (shorter distances) between Eu (III) and the PEP ligands, as well as in the realization of stronger inter- and intramolecular π–π stacking interactions. As a consequence, the Eu(PEP)_2_Cl_3_ shows higher stability in solid state than the Eu(PEP)_2_(NO_3_)_3_ complex. On the other hand, the introduction of bulkier and bidentate NO_3_^−^ counterions stabilizes the complex in solution and, more precisely, in DCM. Experimental measurements confirm that the PEP ligand acts as an antenna for sensitizing Eu (III) luminescence. In addition, higher coordination number and better shielded Eu (III) ion in Eu(PEP)_2_(NO_3_)_3_ are suggested to cause long lifetimes of the excited state in solid and in solution and higher quantum yield in solution. The theoretical simulations in implicit solvent environment demonstrate the influence of the charged co-ligands on the excited triplet state energy of the PEP ligand and suggest that the nitrate groups are more suitable choice for optimization of the excited state energetics and, hence, the antenna effect.

The work demonstrates that variation in the counter ions may leads to significant changes in the structure and properties of Eu (III) luminescent complexes and present an important factor in the molecular and supramolecular design of such materials. The work demonstrates that variation in the counter ions may leads to significant changes in the structure and properties of Eu (III) luminescent complexes and present an important factor in the molecular and supramolecular design of such materials.

## 4. Materials and Methods

### 4.1. Materials Used

All the chemicals for the experiments were analytical grade. Eu(NO_3_)_3_·6H_2_O and EuCl_3_·6H_2_O were synthesized from Eu_2_O_3_ (99.9%, Sigma-Aldrich Chemie GmbH, Taufkirchen, Germany) by dissolving it in dilute HNO_3_ (puriss. p.a. 67%, Sigma-Aldrich Chemie GmbH, Taufkirchen, Germany) and HCl (ACS reagent, 37%, Sigma-Aldrich Chemie GmbH, Taufkirchen, Germany) respectively, followed by crystallization and recrystallizations. Acetonitrile (≥99.9%, HPLC Gradient grade, Fisher Chemicals, Shanghai, China) and DCM (≥99.9%, HPLC Gradient grade, Fisher Chemicals, Shanghai, China) HPLC grade were the solvents used.

The performed organic reactions were monitored with aluminium sheets supported TLC) (silica or neutral alumina, Sigma-Aldrich), visualized under 254 nm UV light. 1,10-phenanthroline monohydrate (99+%, Alfa Aesar, China Chemie GmbH, Beijing, China)) was purchased and used without further purification. 

### 4.2. Preparation of the Ligand and the Complexes

#### 4.2.1. Synthesis of the 2-(Phenylethynyl)-1,10-phenanthroline Ligand

**Scheme 1:**
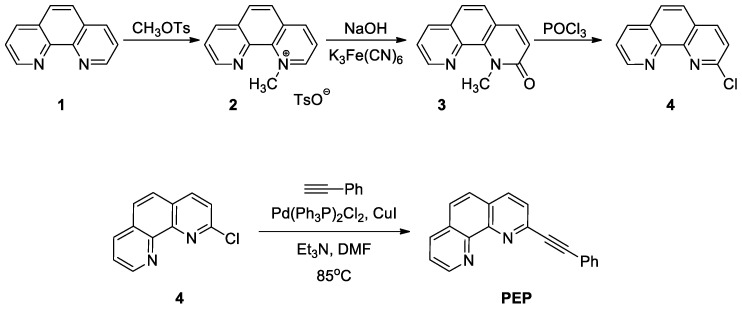
Preparation of PEP ligand.

The ligand 2-(phenylethynyl)-1,10-phenanthroline (PEP) is designed in our lab and synthesized by a multistep protocol starting from pure phenanthroline [49]. The synthetic procedure is represented on Scheme 1.

At the first synthetic step the phenanthroline (1) was alkylated to 1-methyl-1,10-phenanthrolinium tosylate (2) and the resulting salt was deprotonated under oxidizing conditions. Afterwards, 1-methyl-1,10-phenanthrolin-2(1*H*)-one (3) was treated with POCl_3_ to obtain 2-chloro-1,10-phenanthroline (4). Compound (4) was later used in Sonogashira coupling reaction with ethynylbenzene under inert atmosphere in the presence of copper- and palladium-containing catalysts. More details of the synthetic procedure can be found in SM (Synthetic Procedure S1). Similar procedure [50] was used for the synthesis of the corresponding disubstituted phenanthroline. 

The vessel was charged with 1.02 g (4.75 mmol) 2-chloro-1,10-phenanthroline, 0.028 g (0.04 mmol) Pd(Ph_3_P)_2_Cl_2_, 0.030 g (0.158 mmol) copper (I) iodide, 4.5 mL DMF and then was purged with argon. In the next step, 0.626 mL (0.582 g, 5.7 mmol) ethynylbenzene and 1.325 mL (0.962 g, 9.5 mmol) trimethylamine were added and the vessel was purged with argon again. The resulting dark-red reaction mixture was heated to 85 °C and stirred for 21 h. The volatiles were removed under low pressure. The resulting dark residue was dissolved in ethyl acetate and washed with saturated aqueous solution of sodium chloride (5 × 5 mL) and then with 5 mL of pure water. After drying with sodium sulphate, solvent was removed under reduced pressure. The crude product was absorbed on 3 g of silica and was purified by column flash chromatography on silica using as eluent hexanes/DCM/ethyl acetate mixtures with increasing polarity (hexanes, hexanes:DCM = 1:1, pure DCM, DCM:ethyl acetate = 1:1, pure ethylacetate). Yield: 0.96 g (72%) slowly solidifying beige colored oil. 

^1^H NMR (500 MHz, CDCl_3_) δ 9.23 (dd, J = 4.3, 1.6 Hz, 1H, ***H9****-phenanthroline*), 8.23 (dd, J = 8.0, 1.5 Hz, 1H, ***H7****-phenanthroline*), 8.20 (d, J = 8.2 Hz, 1H, ***H4****-phenanthroline*), 7.83 (d, J = 8.2 Hz, 1H, ***H3****-phenanthroline*), 7.77 (d, J = 8.8 Hz, 1H, ***H6****-phenanthroline*), 7.75 (d, J = 8.8 Hz, 1H, ***H5****-phenanthroline*), 7.71–7.66 (m, 2H, ***H2,H6****-phenyl*), 7.63 (dd, J = 8.0, 4.3 Hz, 1H, ***H8****-phenanthroline*), 7.41–7.36 (m, 3H, ***H3***,***H5**-phenyl*, ***H4****-phenyl*).

^13^C NMR (126 MHz, CDCl_3_) δ 150.61 (**C9**-phenanthroline), 146.38 (**^4^C11a-**phenanthroline), 145.70 (**^4^C10a**-phenanthroline), 143.73 (**^4^C2**-phenanthroline), 136.02 (**C7**-phenanthroline), 136.00 (**C4**-phenanthroline), 132.24 (**C2,C6**-phenyl), 129.03 (**C4**-phenyl), 128.92 (**^4^C6a**-phenanthroline), 128.41 (**C3,C5**-phenyl), 127.53 (**^4^C4a**-phenanthroline), 127.05 (**C6**-phenanthroline), 126.57 (**C3**-phenanthroline), 126.15 (**C5**-phenanthroline), 123.23 (**C8**-phenanthroline), 122.52 (**^4^C1**-phenyl), 90.41 (C≡**C**-phenyl), 90.06 (**C**≡C-phenyl).

#### 4.2.2. Synthesis of the Eu (III) Complexes with PEP 

The Eu(PEP)_2_Cl_3_ complex was prepared by mixing at stirring of a hot suspension (80 °C) of EuCl_3_·6H_2_O (0.328 mmol) in acetonitrile, with a hot solution of the ligand 2-(phenylethynyl)-1,10-phenantroline (0.722 mmol) in acetonitrile. The same procedure was applied for the complex Eu(PEP)_2_(NO_3_)_3_ but half of the quantities of the initial reagents Eu(NO_3_)_3_·5H_2_O (0.167 mmol) and the ligand (0.348 mmol) were used. In both syntheses the molar ratio metal to ligand about 1:2 was kept. A slight excess of the ligand was applied because of the solvent traces included.

Afterwards, the mixtures obtained were heated up to 80 °C and stirred for 7 h. At this stage, precipitation was formed and kept for 12 h in the liquid. Finally, the precipitates were filtrated, washed three times with acetonitrile and dried at room temperature. Out of the powder samples obtained single crystals of the complexes in acetonitrile media were formed.

### 4.3. Characterization

The data set was collected using a Bruker D8 Venture diffractometer with a microfocus sealed tube and a Photon II detector. Monochromated MoKα radiation (λ = 0.71073 Å) was used. Data were collected at 133(2) K and corrected for absorption effects using the multi-scan method. The structure was solved by direct methods using SHELXT [51]) and was refined by full matrix least squares calculations on F2 (SHELXL2018 [52]) in the graphical user interface Shelxle [53]. The lanthanide content of the complexes was determined thermogravimetrically by calcination of the complex up to 1300 °C for 4h, taking into account that the powder residue formed was Eu_2_O_3_. The mass of residue was measured by a digital analytical weighing scale with 1 × 10^−5^ g accuracy. DTA/TG were carried out with LABSYSTM Evo-1600, Setaram, Frannce. The infrared spectral analysis ware carried out on a FT-IR Nicolet 6700-Thermo Scientific in KBr pellets. Absorption spectra were obtained with an Evolution 300 UV/VIS spectrometer (Thermo Scientific, Dreieich, Germany) with integration sphere for solid state samples. NMR spectra were recorded on a Bruker Avance III 500 spectrometer at 500 MHz for ^1^H and 125.7 MHz for ^13^C. Lifetime measurements and photoluminescence measurements of the complexes were made on a Cary Eclipse spectrometer with a xenon lamp as excitation source, as well as on a N-400M fluorescent microscope. The quantum yields and photoluminescence measurements are performed at FluoroLog3-22, Horiba JobinYvon equipped with an integration sphere. 

### 4.4. Computational Protocol

Last decades, it has been proved that the DFT/TD-DFT approach is successful for investigation of the structure and spectroscopic properties even of very large complexes containing rare earth elements [54,55,56]. The advantage of the DFT/TF-DFT approach is that it is reasonable time-consuming and requires relatively low computational cost, while its disadvantage is that the results are quantitatively dependent on the choice of the DFT functional. The computational protocol for our study is taken from the recent papers of Georgieva et al. devoted to structurally similar Eu (III) complexes [32]. The ground state geometry of all complexes and ligands were optimized with the ωB97xD functional [57]. Brief discussion on the choice of the DFT functional, as well as on the advantage and limitation of the computational protocol can be found in SM (Discussion S1) [32,58]. For all non-metal elements 6-31G* was used as a basis set. The initial coordinates used for geometry optimizations of the complexes were taken from the corresponding crystallographic data. The electronic shell of europium was described by using the MWB52 basis set, as well as the relativistic effective core potentials MWB52 optimized by Stuttgart-Dresden group [59]. Afterwards, all structures were subject to frequency calculations in order to confirm that they represent minima on the potential energy surface. The absorption spectra, as well as the excited state geometries of the complexes and ligands were obtained at the TD-DFT level of theory within the TDA (Tamm-Dancoff approximation) [60] with the same basis sets and pseudopotentials. The excited states of interest—the first singlet (S_1_) and the first triplet (T_1_) excited states were also optimized. The calculations for ground and excited states were performed in vacuum, as well as in solvents like acetonitrile and dichloromethane. The solvent effects were taken into account by using the polarizable continuum model (PCM) in its integral equation formalism variant (IEFPCM) [61]. All calculations have been performed with Gaussian 09 [62].

## Figures and Tables

**Table 1:** Selected geometric parameters from single crystal measurements: bond lengths, R [Å] and dihedral angles θ [^o^] for complexes Eu(PEP)_2_Cl_3_ and Eu(PEP)_2_(NO_3_)_3_. For simplicity, the original labels of some atoms are omitted and replaced by chemically intuitive ones: N—is the nitrogen atom from the non-functionalized phenanthroline ring, N^≡^—is the nitrogen atom from the phenanthroline ring functionalized with -C≡C-C_6_H_5_ substituent, Cl^top^ and O^top^—are the counterions lying on the top of the N-Eu-N line.

	Eu(PEP)_2_Cl_3_		Eu(PEP)_2_(NO_3_)_3_	
R [Å]	Eu–N	2.5320(13)	Eu–N	2.6006(15)
	Eu–N^≡^	2.5822(14)	Eu–N^≡^	2.6253(15)
	Eu–N	2.5400(14)	Eu–N	2.5958(15)
	Eu–N^≡^	2.5806(12)	Eu–N^≡^	2.6542(15)
	Eu1–Cl1	2.6930(5)	Eu–O1	2.5247(14)
	Eu-Cl^top^	2.6893(7)	Eu–O2	2.4813(13)
	Eu–Cl2	2.6744(5)	Eu–O^top^	2.5273(14)
			Eu–O^top^	2.5164(14)
			Eu–O7	2.5371(14)
			Eu–O8	2.5022(13)
Θ [^o^]	Cl1–Eu–Cl2	165.30(3)	O1–Eu–O2	51.07(4)
	Cl2–Eu–Cl^top^	82.71(2)	O^top^–Eu–O^top^	50.60(5)
	Cl1–Eu-Cl^top^	82.59(2)	O7–Eu–O8	50.74(4)
	N–Eu–N	64.30(4)	N–Eu–N	63.42(5)
	N^≡^–Eu–N	64.45(4)	N–Eu–N^≡^	63.31(5)

**Table 2:** Optimized bond lengths [Å] for ground state of complexes Eu(PEP)_2_Cl_3_ and Eu(PEP)_2_(NO_3_)_3_. DFT results obtained in acetonitrile (a) and dichloromethane (d) with the ωB97xD method.

	Eu(PEP)_2_Cl_3_			Eu(PEP)_2_(NO_3_)_3_	
	(d)	(a)		(d)	(a)
Eu–N	2.651	2.647	Eu–N	2.627	2.628
Eu–N^≡^	2.664	2.654	Eu–N^≡^	2.685	2.675
Eu–N	2.651	2.648	Eu–N	2.627	2.628
Eu–N^≡^	2.664	2.653	Eu–N^≡^	2.685	2.675
Eu–Cl^≡^	2.778	2.791	Eu–O	2.548	2.554
Eu–Cl^top^	2.744	2.759	Eu–O	2.539	2.542
Eu–Cl	2.778	2.791	Eu–O^top^	2.511	2.519
			Eu–O^top^	2.511	2.519
			Eu–O	2.548	2.554
			Eu–O	2.539	2.542

**Table 3:** Luminescence parameters of Eu(PEP)_2_Cl_3_ and Eu(PEP)_2_(NO_3_)_3_.

Sample	Life Time in µs	QY%
Eu(PEP)_2_Cl_3_ *solid state*	460 ± 11	-
Eu(PEP)_2_(NO_3_)_3_ *solid state*	1337 ± 2	-
Eu(PEP)_2_Cl_3_ in *solution/acetonitrile*	703 ± 1	8.8 ± 0.5
Eu(PEP)_2_(NO_3_)_3_ in *solution/acetonitrile*	1393 ± 25	9.6 ± 0.5
Eu(PEP)_2_Cl_3_ in *solution/DCM*	572 ± 4	1.4 ± 0.2
Eu(PEP)_2_(NO_3_)_3_ in *solution/DCM*	1665 ± 35	28.1 ± 0.5

**Table 4:** Vertical excitation and emission energies, E_v_ [eV], and oscillator strength, *f*, as well as 0–0 emission energies, E_0__–0_, for the PEP ligand and complexes: singlet ground state (S_0_), first singlet excited state (S_1_) and first triplet excited state (T_1_). TD-DFT/TDA results obtained in acetonitrile (a) and dichloromethane (d) with the ωB97xD method.

	Eu(PEP)_2_Cl_3_		Eu(PEP)_2_(NO_3_)_3_		PEP	
	(d)	(a)	(d)	(a)	(d)	(a)
E_v_ (S_0_→S_1_)	4.10	4.11	4.23	4.23	4.17	4.18
*f* (S_0_→S_1_)	0.1313	0.1193	0.0577	0.0528	1.1996	1.1412
E_v_ (S_0_→T_1_)	3.10	3.10	3.16	3.16	3.02	3.02
E_v_ (T_1_→S_0_)	2.14	2.13	2.35	2.35	2.29	2.29
E_0–0_ (T_1_→S_0_)	2.45	2.45	2.62	2.62	2.67	2.67

Second excited state is reported since S_1_ (3.52 eV) is ‘dark’ with *f* = 0.0000.

## Data Availability

Crystallographic data have been deposited at Cambridge Crystallographic Data Center under the reference number: 2117932 and 2117935.

## Supplementary Materials

The following are available online, Figure S1: TG/DTA curves of (a) Eu(PEP)_2_Cl_3_ and (b) Eu(PEP)_2_(NO_3_)_3_; Figure S2: 1H-NMR spectrum of Eu(PEP)2Cl3 complex in MeOD; Figure S3: ^13^C-NMR spectrum of Eu(PEP)_2_Cl_3_ complex in MeOD; Table S1: Experimentally observed and simulated vibrational frequencies *ω*[cm^−1^] for the PEP ligand and the complexes, as well as an assignment of the IR bands. The spectra were measured in KBr. The Calculations were done in vacuum with the ωB97xD method, MWB52 basis set and pseudopotentials for Eu, and 6-31G* for all non-metal atoms. The calculated vibrational frequencies were scaled by 0.949, which is a scaling factor for ωB97xD/6-31G* level of theory; Table S2: Intra- and Intermolecular distances of the complexes; Figure S4: Absorption spectra of PEP ligand in: (a) solid state and (b) in acetonitrile solution; Figure S5: Normalized absorption spectra of ligand, Eu(PEP)_2_Cl_3_ and Eu(PEP)_2_(NO_3_)_3_ in (a) acetonitrile and (b) DCM solution; Figure S6: Excitation and emission spectra of PEP ligand in solid state; Figure S7: Excitation and emission spectra of PEP ligand in (a) DCM solution and (b) acetonitrile solution; Figure S8: Emission spectra in DCM of (a) Eu(PEP)_2_Cl_3_ and (b) Eu(PEP)_2_(NO_3_)_3_; Table S3: Free energy, ΔG [kcal/mol], for the reaction of the complex formation: Eu(H_2_O)_4_A_3_ + 2PEP = 4H_2_O + Eu(PEP)_2_A_3_, where A= Cl^−^ or NO_3_^−^. Results obtained with wB97xD method in acetonitrile (a) and dichloromethane (d). G=G(Eu(PEP)_2_A_3_) + 4G(H_2_O) − G(Eu(H_2_O)_4_A_3_) − 2G(PEP); Synthetic Procedure S1: Details on Scheme 1 from manuscript.; Figure S9: 1-methyl-1,10-phenanthrolin-1-ium tosylate (2); Figure S10: 1-methyl-1,10-phenanthrolin-2(1H)-one (3); Figure S11: 2-chloro-1,10-phenanthroline (4); Discussion S1. Choice of DFT functional, as well as discussion on the advantages and limitation of the computational protocol used.; Table S4: Relative luminescence intensity.

## Sample Availability

Samples of the compounds in this article are not available from the authors.
