# Supplementary material for: Luminescent Complexes of Europium (III) with 2-(Phenylethynyl)-1,10-phenanthroline: The Role of the Counterions
Source: Molecules. 2021 Nov 30;26(23):7272. doi: 10.3390/molecules26237272 (PMC8658859; doi:10.3390/molecules26237272)
Supplement: Supplementary file 1 [file molecules-26-07272-s001.zip › molecules-1457810-supplementary.pdf]

Luminescent Complexes of Europium (III) with 2-(Phenylethynyl)-1,10-phenanthroline: The Role of the Counterions

Denitsa Elenkova[1,*], Rumen Lyapchev[1], Julia Romanova[1], Bernd Morgenstern[2], Yana Dimitrova[1], Deyan Dimov[3], Martin Tsvetkov[1], Joana Zaharieva[1]

[1] Faculty of Chemistry and Pharmacy, Sofia University, 1164 Sofia, Bulgaria
[2] Saarland University, 66123 Saarbrücken, Germany
[3] Institute of Optical Materials and Technologies, Bulgarian Academy of Science, 1113 Sofia, Bulgaria
* Correspondence: d.elenkova@chem.uni-sofia.bg; Tel.: +359-2-8161325

Contents

Figure S1. TG/DTA curves of (**a**) $Eu(PEP)_2Cl_3$ and (**b**) $Eu(PEP)_2(NO_3)_3$.

Figure S2. ^{1}H-NMR spectrum of $Eu(PEP)_2Cl_3$ complex in MeOD.

Figure S3. ^{13}C-NMR spectrum of $Eu(PEP)_2Cl_3$ complex in MeOD.

Table S1: Experimentally observed and simulated vibrational frequencies ω [cm^{-1}] for the PEP ligand and the complexes, as well as an assignment of the IR bands. The spectra were measured in KBr . The Calculations were done in vacuum with the ωB97xD method, MWB52 basis set and pseudopotentials for Eu, and 6-31G* for all non-metal atoms. The calculated vibrational frequencies were scaled by 0.949, which is a scaling factor for ωB97xD/6-31G* level of theory.

Table S2: Intra- and Intermolecular distances of the complexes.

Figure S4. Absorption spectra of PEP ligand in: (**a**) solid state and (**b**) in acetonitrile solution.

Figure S5. Normalized absorption spectra of ligand, $Eu(PEP)_2Cl_3$ and $Eu(PEP)_2(NO_3)_3$ in (**a**) acetonitrile and (**b**) DCM solution.

Figure S6. Excitation and emission spectra of PEP ligand in solid state.

Figure S7. Excitation and emission spectra of PEP ligand in (**a**) DCM solution and (**b**) acetonitrile solution.

Figure S8. Emission spectra in DCM of (**a**) $Eu(PEP)_2Cl_3$ and (**b**) $Eu(PEP)_2(NO_3)_3$.

Table S3. Free energy, G [kcal/mol], for the reaction of the complex formation: $Eu(H_2O)_4A_3 + 2PEP = 4H_2O + Eu(PEP)_2A_3$, where A= Cl^- or NO_3^-. Results obtained with wB97xD method in acetonitrile (a) and dichloromethane (d). $G = G(Eu(PEP)_2A_3) + 4G(H_2O) - G(Eu(H_2O)_4A_3) - 2G(PEP)$.

Synthetic procedure S1: Details on Scheme 1 from manuscript.

Figure S9. 1-methyl-1,10-phenanthrolin-1-ium tosylate (2).

Figure S10. 1-methyl-1,10-phenanthrolin-2(1*H*)-one (3).

Figure S11. 2-chloro-1,10-phenanthroline (4).

Discussion S1. Choice of DFT functional, as well as discussion on the advantages and limitation of the computational protocol used.

Table S4. Relative luminescence intensity.

References

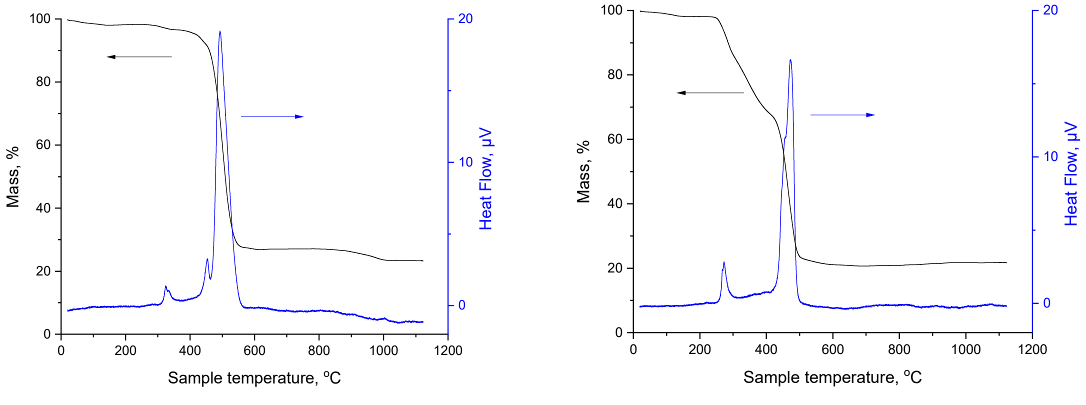

(**a**) (**b**)

Figure S1. TG/DTA curves of (**a**) $Eu(PEP)_2Cl_3$ and (**b**) $Eu(PEP)_2(NO_3)_3$

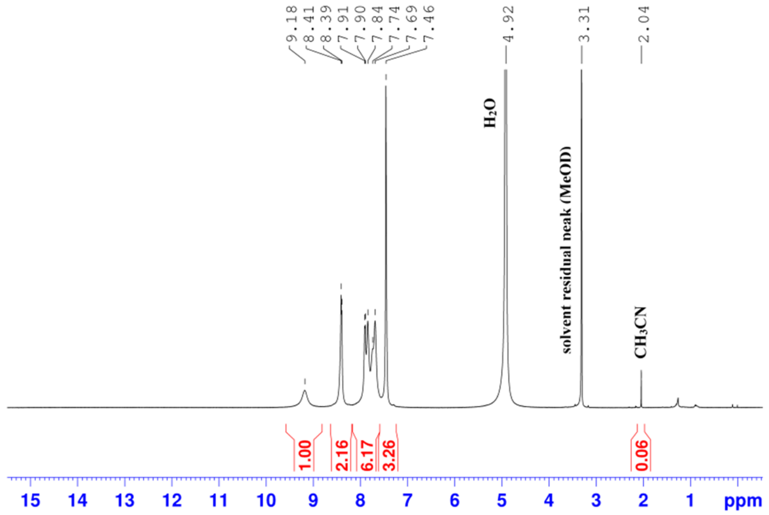

Figure S2: ^{1}H-NMR spectrum of $Eu(PEP)_2Cl_3$ complex in MeOD.

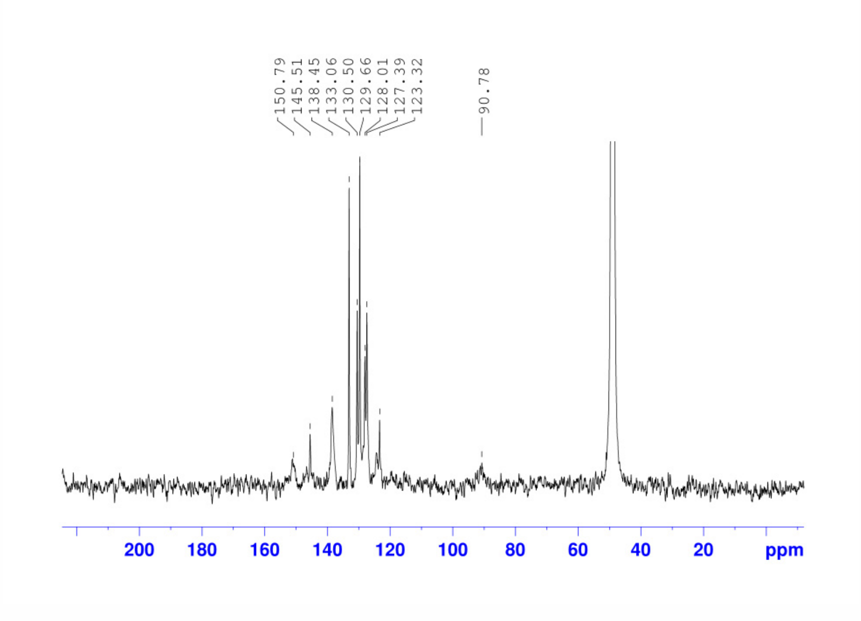

Figure S3. ^{13}C-NMR spectrum of Eu(PEP)$_2$Cl$_3$ complex in MeOD.

Table S1: Experimentally observed and simulated vibrational frequencies ω [cm^{-1}] for the PEP ligand and the complexes, as well as an assignment of the IR bands. The spectra were measured in KBr. The Calculations were done in vacuum with the ω=B97xD method, MWB52 basis set and pseudopotentials for Eu, and 6-31G* for all non-metal atoms. The calculated vibrational frequencies were scaled by 0.949, which is a scaling factor for ωB97xD/6-31G* level of theory.

**Eu(PEP)$_2$Cl$_3$ ωExp.***	**Eu(PEP)$_2$Cl$_3$ ωTh.***	**Eu(PEP)$_2$(NO$_3$)$_3$ ωExp.***	**Eu(PEP)$_2$(NO$_3$)$_3$ ωTh.***	**Assignment****	**PEP ωExp.**	**PEP ωTh.**	**Assignment****
3010 - 3110	3051-3102	3010-3110	3054 - 3100	ϑ (CH)	2950-3120	3035-3093	ϑ (CH)
2207 (0)	2267(-11)	2208	2271	ϑ (-C≡C-)	2207	2256	ϑ (-C≡C-)
1622 (-9)	1634 (-8)	hidden [a]	1634	ϑ (CC) + δ (CCH)	1613	1626	ϑ (CC) + δ (CCH)
		1623	1624	ϑ(N-O)			
1600 (-5)	1618 (-4)	hidden [a]	1619	ϑ (CC) + ϑ (CN)+δ (CCH)	1595	1614	ϑ (CC) + ϑ (CN) + δ (CCH)
1586 (-4)	1602 (-2)	hidden [a]	1603	ϑ (CC) + ϑ (CN) + δ (CCH)	1582	1600	ϑ (CC) + ϑ (CN) + δ (CCH)
		1592	1600;1590	ϑ(N-O)			
1562 (-16)	1569(-18)	1563 (-17)	1571 (-20)	ϑ (CC) + ϑ (CN) + δ (CCH)	1546	1551	ϑ (CC) + ϑ (CN) + δ (CCH)
1509 (-6)	1513 (-11)	1509 (-6)	1510 (-8)	ϑ (CC) + δ (CCH)	1503	1502	ϑ (CC) + δ (CCH)
1489 (-6)	1491 (-6)	1489 (-6)	1492(-7)	ϑ (CC) + δ (CCH)	1483	1485	ϑ (CC) + δ (CCH)
1450 (-7)	1447 (-10)	1454 (-12)	1447 (-10)	ϑ (CC) + δ (CCH)	1443	1437	ϑ (CC) + δ (CCH)
1417 (-15)	1408 (-10)	1417 (-15)	1407 (-11)	ϑ (CC) + δ (CCH)	1402	1398	ϑ (CC) + δ (CCH)
1390 (-4)	1388 (-8)	1384	1389 (-9)	ϑ (CN) + ϑ (CC) + δ (CCH)	1386	1380	ϑ (CN) + ϑ (CC) + δ (CCH)
		1334 (sh) [a]	1342; 1334	ϑ(N-O)			

-		-			1326	1321	ϑ (CN) + ϑ (CC) + δ (CCH)
		1312	1319	ϑ(N-O)			
1304 (-5)	1300 (-8)	1310 (-11)	1304	ϑ (CN) + ϑ (CC) + δ (CCH)	1299 (sh) [a]	1292 (sh) [a]	ϑ (CN) + ϑ (CC) + δ (CCH)
1292 (-6)	1287 (-8)	1292 (-6)	1281(-2)	ϑ (CC) + δ (CCH)	1286	1279	ϑ (CC) + δ (CCH)
1250-1000	1250-1000	1250-1000	1250-1000	δ (CCH)	1250-1000	1250-1000	δ (CCH)
			1064; 1060	δ (O-N-O)			
1000-500	1000-500	1000-500	1000-500	η and δ (CCC/CNC)	1000-500	1000-500	η and δ (CCC/CNC)
		782	795	η (NO_3^-)			
		740; 724; 691	734; 727; 724; 694; 692	δ (O-N-O)			
-		-			627	613	δ (CNC) + δ (CCC)

*The data in the brackets represent the vibrational frequency difference [cm^{-1}] between the PEP and the complex: $\Delta\omega = \omega_{PEP} - \omega_{complex}$

** ϑ – stretching; δ – bending in plane; η -bending out of plane

[a] hidden – the identification of the peak is prohibited due to its overlap with the peaks from the ϑ(N-O) group. Such overlap leads to the observation of broad IR peaks with maxima around 1592 and 1623 cm^{-1}

[b] sh – showder

Table S2: Intra- and Intermolecular distances of the complexes.

Eu(PEP)$_2$Cl$_3$				
**Number of centroid (Cg)**	**Atom Labels**			
Cg(4)	N4→C26→C27→C28→C29→C30 (6-Membered Ring)			
Cg(5)	C35→C36→C37→C38→C39→C40 (6-Membered Ring)			
Cg(6)	C15→C16→C17→C18→C19→C20 (6-Membered Ring)			
Cg(7)	C24→C25→C26→C27→C31→C32 (6-Membered Ring)			
Cg(8)	C4→C5→C6→C7→C11→C12 (6-Membered Ring)			
**π···π interaction**	**Type**	Cg···Cg(Å)*	Slippage d[a](Å)*	α(°)*
Cg(4)···Cg(7)	Inter-	3.5071(9)	0.616/0.715	1.64(7)
Cg(4)···Cg(6)	Intra-	3.7873(10)	1.258/1.784	10.31(8)
Cg(5)···Cg(8)	Intra-	3.6598(10)	1.045/1.262	8.37(8)
Eu(PEP)$_2$(NO$_3$)$_3$				
**Number of centroid (Cg)**	**Atom Labels**			
Cg(1)	N1→C1→C2→C3→C4→C5 (6-Membered Ring)			
Cg(3)	N3→C21→C22→C23→C24→C25 (6-Membered Ring)			
Cg(5)	C4→C5→C6→C7→C11→C12 (6-Membered Ring)			
Cg(6)	C15→C16→C17→C18→C19 →C20(6-Membered Ring)			

Cg(7)	C24→C25→C26→C27→C31→C32 (6-Membered Ring)			
Cg(8)	C35→C36→C37→C38→C39→C40 (6-Membered Ring)			
**π···π interaction**	**Type**	Cg···Cg(Å)*	Slippage d[a](Å)*	α(°)*
Cg(1)···Cg(7)	Inter-	3.9423(10)	1.638/1.610	1.73(9)
Cg(1)···Cg(8)	Intra-	3.7704(12)	1.380/1.742	6.15(10)
Cg(3)···Cg(5)	Inter-	3.9522(11)	1.502/1.610	3.95(9)
Cg(3)···Cg(6)	Intra-	3.7584(12)	1.435/1.912	8.29(9)

*Cg···Cg – distance from centroid to centroid, Slippage d[a] – distance between Cg(I) and perpendicular projection of Cg(J) on Ring I ; α – dihedral angle between planes of interacting Rings.

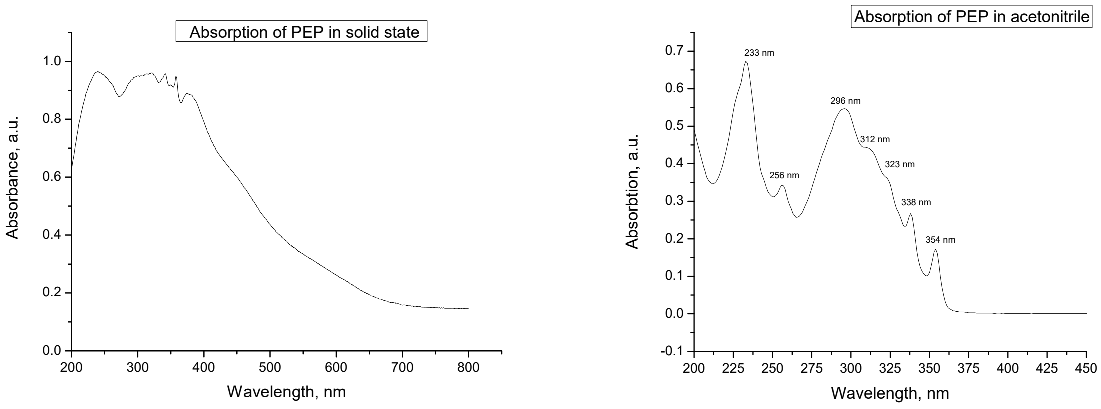

(**a**) (**b**)

Figure S4. Absorption spectra of PEP ligand in: (**a**) solid state and (**b**) in acetonitrile solution.

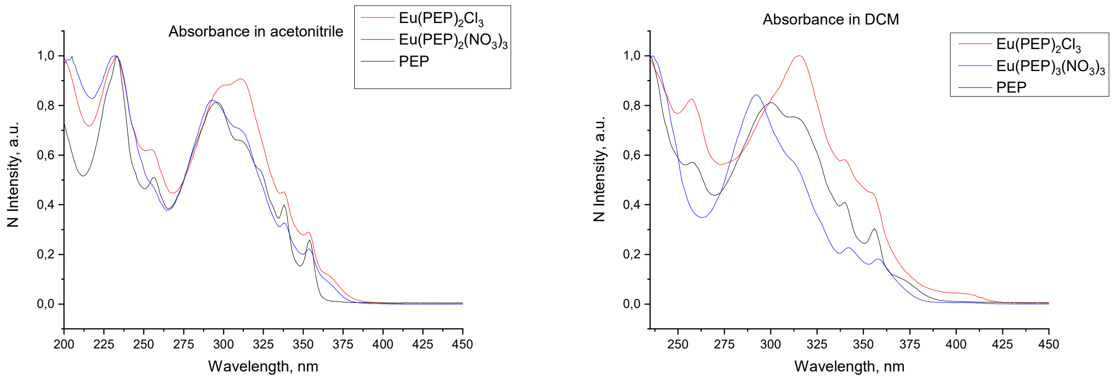

(**a**) (**b**)

Figure S5. Normalized absorption spectra of ligand, $Eu(PEP)_2Cl_3$ and $Eu(PEP)_2(NO_3)_3$ in (**a**) acetonitrile and (**b**) DCM solution.

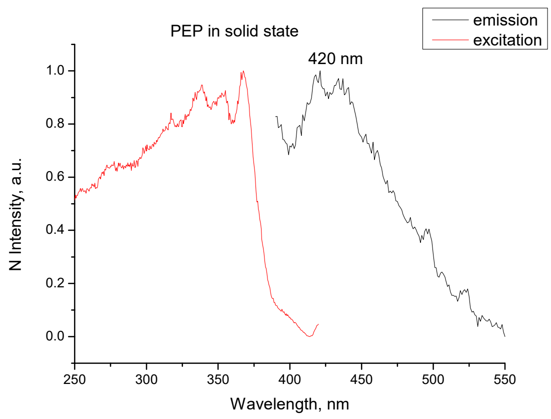

Figure S6. Excitation and emission spectra of PEP ligand in solid state.

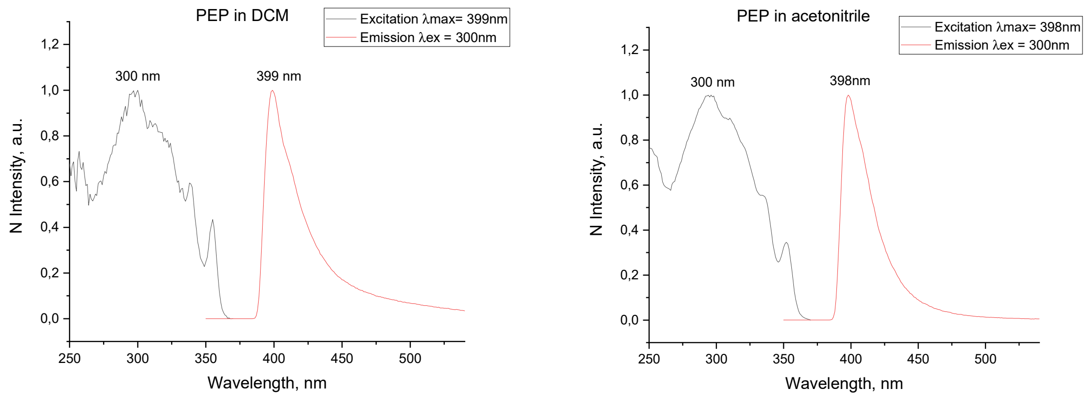

(**a**) (**b**)

Figure S7. Excitation and emission spectra of PEP ligand in (**a**) DCM solution and (**b**) acetonitrile solution.

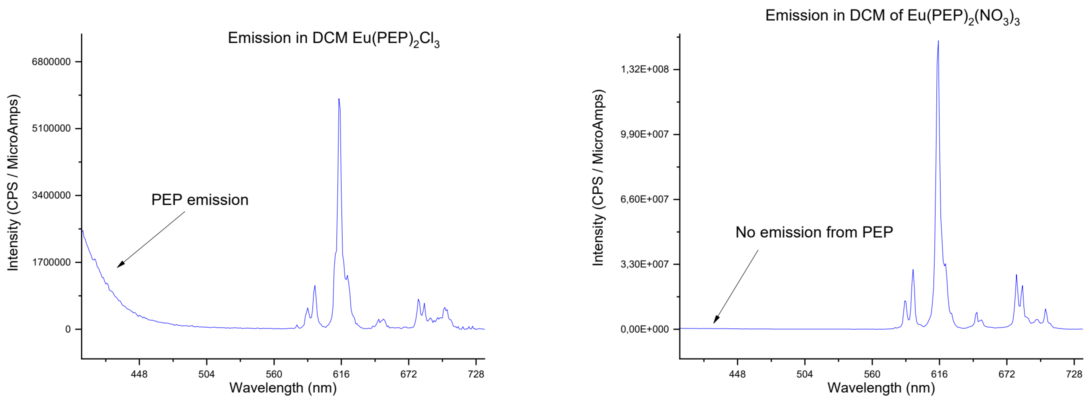

(**a**) (**b**)

Figure S8. Emission spectra in DCM of (**a**) $Eu(PEP)_2Cl_3$ and (**b**) $Eu(PEP)_2(NO_3)_3$

Table S3. Free energy, ΔG [kcal/mol], for the reaction of the complex formation: $Eu(H_2O)_4A_3 + 2PEP = 4H_2O + Eu(PEP)_2A_3$, where A= Cl^- or NO_3^-. Results obtained with ωB97xD method in acetonitrile (a) and dichloromethane (d). $\Delta G = G(Eu(PEP)_2A_3) + 4G(H_2O) - G(Eu(H_2O)_4A_3) - 2G(PEP)$.

	ΔG	
	(d)	(a)

$Eu(PEP)_2Cl_3$	-14.95	-15.87
$Eu(PEP)_2(NO_3)_3$	-19.76	-19.93

Synthetic procedure S1: Details on Scheme 1 from manuscript

Phenanthroline monohydrate (1) (15.1 g, 76.2 mmol) and methyl tosylate (28.4 g, 152.5 mmol) were dissolved in 45 ml of acetonitrile under inert atmosphere and the solution was refluxed for 3 hours. The cooled mixture was poured onto 250 ml of THF. The resulting suspension was stored in a freezer for 12 hours. The white crystalline solid formed was filtered and washed with THF and diethyl ether. The product was dried in air. Yield: 26.16 g (94%) of colorless crystals.

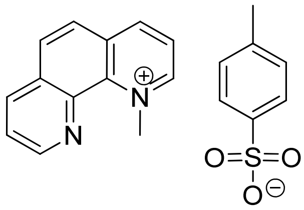

Figure S9. 1-methyl-1,10-phenanthrolin-1-ium tosylate (2).

The ^{1}H and ^{13}C NMR spectra of the compound were identical to those described in the literature [1].

Three solutions were prepared by dissolving tosylate salt (2) (25.2 g, 68.77 mmol) in 250 ml of water (solution A), NaOH (50 g, 1.25 mol) in 250 ml of water (solution B) and $K_3[Fe(CN)_6]$ (50 g, 151.8 mmol) in 250 ml of water (solution C). Solution C was cooled on an ice bath in a beaker. Solution A and solution B were simultaneously added dropwise to solution C for 20 minutes while the mixture was constantly vigorously stirred by a mechanical stirrer. After all the solutions were added to the beaker, the reaction mixture was left to warm to room temperature and then stirred for 1 hour. After that the formed yellow suspension was cooled on an ice bath and collected by vacuum filtration. The crude product was washed with water, dried and dissolved in CH_2Cl_2. Traces of water in the solution were removed by drying with anhydrous Na_2SO_4. The organic solvent was removed under reduced pressure. Yield: 13.42 g (93%) of pale yellow powder.

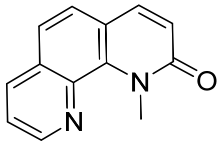

Figure S10. 1-methyl-1,10-phenanthrolin-2(1H)-one (3).

The ^{1}H and ^{13}C NMR spectra of the compound were identical to those described in the literature [Ref S1].

1-methyl-1,10-phenanthrolin-2(1H)-one (3) (13.42 g, 63.84 mmol) was mixed with 65 ml $POCl_3$ in a flask under solvent free conditions, equipped with a condenser connected to an oil bubbler. The mixture was gently refluxed for 5 hours. The excess $POCl_3$ was removed under reduced pressure and 100 ml of water were added to the crude product. The suspension was neutralized with K_2CO_3 and the formed crude mixture was extracted with $CHCl_3$. The combined organic layers were dried with Na_2SO_4 and the solvent was removed under reduced pressure. Yield: 9.9 g (72%) of brown powder.

For applying the 2-chloro-1,10-phenanthroline (4) in Sonogashira coupling, the brown powder was purified by column chromatography and obtained in the form of white crystals.

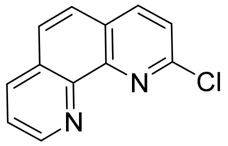

Figure S11. 2-chloro-1,10-phenanthroline (4).

The ^{1}H and ^{13}C NMR spectra of the compound were identical to those described in the literature [1].

Discussion S1. Choice of DFT functional.

Here, it should be briefly noted that our preliminary investigations showed that the TD-DFT/TDA approach with the ωB97xD functional overestimates the energy of the $S_0 \rightarrow S_1$ transition and that more accurate prediction of the $S_0 \rightarrow S_1$ energy can be obtained at the PBE0 level. However, the PBE0 functional significantly underestimate the energy of the T_1 state, which is a key player in the energy transfer. Therefore, we selected the ωB97xD functional for qualitative interpretation of the excited state energetics in the complexes.

It is important to stress that the oscillator strengths of the electron transitions involving triplet excited states obtained with the conventional TD-DFT approach are always spin forbidden, which is a limitation of the method used here. In reality, the triplet transitions in europium complexes can be observed due to the existence of spin-orbit coupling (SOC) effects conditioned by the presence of the rare earth ion. Therefore, better description of the optical properties of the complexes is expected when SOC are taken into account in the TD-DFT calculations. For example, in their recent paper Tanner et al. investigated the effect of the inclusion of the SOC in the TD-DFT calculations on the theoretically predicted absorption spectra for the complex between Eu3+ and non-substituted phenanthroline. The authors observed an increase in the oscillator strength of the triplet transitions when the SOC effects are taken into account [Ref. S2]. Moreover, the spin-orbit coupling effects in rare-earth complexes have direct impact on the ligand-to-metal energy transfer rates [Ref. S3]. However, since our discussion here is based on the change in the excitation/de-excitation energies, we believe that the omittement of the SOC effects in the TD-DFT calculations is a good compromise between accuracy and computational resources.

Table S4. Relative luminescence intensity*

Eu 1 in solid state			*Eu1 acetonitrile solution*			*Eu1 dichloromethane solution*		
$^5D_0 \rightarrow ^7F_J$	*Norm. to* $^5D_0 \rightarrow ^7F_1$	*% of total emission*	$^5D_0 \rightarrow ^7F_J$	*Norm. to* $^5D_0 \rightarrow ^7F_1$	*% of total emission*	$^5D_0 \rightarrow ^7F_J$	*Norm. to* $^5D_0 \rightarrow ^7F_1$	*% of total emission*
0→0	*0.14*	*1.38*	*0→0*	*0.27*	*1.95*	*0→0*	*0.04*	*0.41*
0→1	*1.00*	*10.01*	*0→1*	*1.00*	*7.16*	*0→1*	*1.00*	*11.57*
0→2	*7.19*	***71.99***	*0→2*	*11.37*	***81.39***	*0→2*	*5.58*	***64.54***

0→3	*0.25*	*2.54*	*0→3*	*0.34*	*2.42*	*0→3*	*0.33*	*3.76*
0→4	*1.41*	*14.08*	*0→4*	*0.99*	*7.08*	*0→4*	*1.71*	*19.73*
Total/0→1	*9.99*		*Total/0→1*	*13.97*		*Total/0→1*	*8.65*	
Eu2 in solid state			*Eu2 acetonitrile solution*			*Eu2 dichloromethane solution*		
$^5D_0\rightarrow{}^7F_J$	*Norm. to* $^5D_0\rightarrow{}^7F_1$	*% of total emission*	$^5D_0\rightarrow{}^7F_J$	*Norm. to* $^5D_0\rightarrow{}^7F_1$	*% of total emission*	$^5D_0\rightarrow{}^7F_J$	*Norm. to* $^5D_0\rightarrow{}^7F_1$	*% of total emission*
0→0	*0.01*	*0.22*	*0→0*	*0.02*	*0.20*	*0→0*	*0.02*	*0.19*
0→1	*1.00*	*14.97*	*0→1*	*1.00*	*11.45*	*0→1*	*1.00*	*12.30*
0→2	*3.82*	***57.18***	*0→2*	*5.66*	***64.83***	*0→2*	*5.10*	***62.71***
0→3	*0.30*	*4.41*	*0→3*	*0.37*	*4.19*	*0→3*	*0.36*	*4.42*
0→4	*1.55*	*23.23*	*0→4*	*1.69*	*19.34*	*0→4*	*1.66*	*20.37*
Total/0→1	*6.68*		*Total/0→1*	*8.74*		*Total/0→1*	*8.13*	

*From Figure 8. Normalized to $^5D_0\rightarrow{}^7F_1$ transition.

References

S1. Krapcho, A.P.; Lanza, J.B. Improved Synthesis of 2,9-Dichloro-1,10- phenanthroline. *Org. Prep. Proced. Int.* **2007**, *39*, 603–620.

S2. Zhang, Y.; Thor, W.; Wong, K.-L.; Tanner, P.A. Determination of Triplet State Energy and the Absorption Spectrum for a Lanthanide Complex. *J. Phys. Chem. C* **2021**, *125*, 7022–7033, doi:10.1021/acs.jpcc.1c00158.

S3. Zahariev, T.; Shandurkov, D.; Gutzov, S.; Trendafilova, N.; Enseling, D.; Jüstel, T.; Georgieva, I. Phenanthroline chromophore as efficient antenna for Tb^{3+} green luminescence: A theoretical study. *Dye. Pigment.* **2021**, *185*, 108890, doi:10.1016/j.dyepig.2020.108890.
